# Environmental phenotypes for healthy weight in children using population-based linked environment and health data: a cross-sectional observational study

**DOI:** 10.1016/j.healthplace.2026.103681

**Published:** 2026-07

**Authors:** Jo Davies, Rowena Bailey, Rebecca Pedrick-Case, Gareth Stratton, Theodora Pouliou, Amy Mizen, Hayley Christian, Bryan Boruff, Ronan A. Lyons, Rich Fry, Lucy J. Griffiths

**Affiliations:** a Population Data Science, Swansea University Medical School, Swansea, UK; b Research Centre in Applied Sports, Technology, Exercise and Medicine, Swansea University, Swansea, UK; c Telethon Kids Institute, University of Western Australia, Perth, Western Australia, Australia; d School of Population and Global Health, University of Western Australia, Perth, Western Australia, Australia; e School of Agriculture and Environment, University of Western Australia, Perth, Western Australia, Australia; f School of Public Health and Preventive Medicine, Monash University, Melbourne, Victoria, Australia

## Abstract

**Background:**

Childhood obesity is a major global health challenge, projected to affect one in three children worldwide by 2050. While individual and social factors contribute, increasing evidence highlights the built environment as a key determinant in shaping children's behaviours and weight outcomes. Evidence suggests that neighbourhood design, greenspace access, and food retail availability influence diet and physical activity, but most studies rely on small samples or single-domain measures.

**Methods:**

We linked nationwide geographic information systems (GIS) data describing residential neighbourhoods with objectively measured child weight from a national Welsh surveillance programme for children aged 4–5 years. Using multiple indicators—including housing type, garden size, neighbourhood greenness, walkability, access to recreational spaces, and food outlet density - latent class analysis was used to identify distinct “environmental phenotypes.” Associations between phenotypes and child weight status were examined using logistic regression.

**Results:**

We identified discrete classes of residential environments characterised by varying combinations of built and food environment features. The model with five classes was the best fit overall, with percentage and number of households in each phenotypes: *Rural, spacious and isolated* 14% (24,266), *Suburban* 17% (29,324), *Deprived and underserved* 23% (39,227), *Deprived and well-served* 32% (53,210), and *Dense, coastal and well-connected* 13% (21,762). Children living in *rural, spacious and isolated* neighbourhoods, characterised as those with greater greenspace, private gardens, and walkable layouts, had significantly lower odds of overweight and obesity (OR = 0.89, CI = 0.86-0.93), whereas those in *deprived and well-served* neighbourhoods, characterised by high-density housing areas with limited greenspace and high fast-food outlet density, had elevated risk (OR = 1.09, CI = 1.06-1.13). These associations remained robust after adjustment for area-level deprivation and rurality.

**Conclusion:**

Our findings highlight the importance of the residential environment in early childhood obesity risk. Nationally linked GIS and health data enable robust classification of obesogenic environments, informing urban planning and public health strategies to promote healthier, child-friendly neighbourhoods.

## Highlights

• Nationwide linkage of GIS and health surveillance data to examine residential environments and childhood obesity at a population scale.
• Comprehensive environmental profiling using multiple indicators.
• Novel classification of “environmental phenotypes” capturing complex combinations of built and food environment features.
• Children living in *rural, spacious and isolated* neighbourhoods had significantly lower odds of overweight and obesity.
• Actionable insights for urban planning and public health to design child-friendly, obesity-preventive neighbourhoods.

## 1 Introduction

Childhood obesity is one of the most pressing global health challenges of the 21st century. The global prevalence of overweight and obesity in children and adolescents aged 5-19 rose from 8% in 1990 to 20% in 2022 (Obesity and overweight n). These trends are not only accelerating, but are projected to intensify – by 2050, nearly one in three children worldwide may be living with excess weight (GBD, 2021 Adolescent BMI Collaborators, 2025). The implications of this public health crisis are profound, increasing the risk of early-onset chronic diseases and placing a growing burden on already strained healthcare systems (Ling et al., 2023). While childhood weight status is influenced by a range of factors, growing attention has been directed towards the role of the built environment in shaping children's health behaviours and outcomes.

Early childhood represents a critical period for growth and development, during which patterns of diet, physical activity and weight gain are established. Young children are also more dependent on their immediate residential environment than older children as their access to food, play spaces and transport are determined by their parents or caregivers. As such, a greater understanding of the neighbourhood-level environmental characteristics that influence childhood weight status is essential to inform effective prevention strategies during this critical developmental phase.

Evidence suggests that the built environment - encompassing housing type, neighbourhood design, land use, access to amenities, and foot outlet density - plays a crucial role in influencing children's diet and physical activity and can either promote or inhibit healthy behaviours (Malacarne et al., 2022; Miller et al., 2014; Schalkwijk et al., 2018a; Maitland et al., 2013; Mizen et al., 2018; Mayor, 2014). Well-connected neighbourhoods with pedestrian-friendly routes can encourage active transport and outdoor play. In contrast, unsafe or poorly designed urban areas with limited access to designated greenspace can discourage physical activity, leading children to spend more time indoors engaging in sedentary behaviours (Daniels et al., 2021a; Datar et al., 2013). Several studies have examined these associations in young children (Nordbø et al., 2019; Reimers and Knapp, 2017; Jia et al., 2020; Terrón-Pérez et al., 2021) although evidence specific to the 4-5 year age group remains limited compared with studies of older children. Spence and colleagues (Spence et al., 2008) analysed 501 preschool-aged children in Edmonton, Canada and explored how measures of neighbourhood design related to overweight status, finding modest associations with built environment features in this age group. A systematic review of longitudinal studies across broader age ranges in the United States and in European settings suggest that built and social neighbourhood characteristics such as access to green space, parks, and recreational facilities may have beneficial effects on weight trajectories in children, whereas features of the food environment show mixed associations (Daniels et al., 2021b). Overall, while neighbourhood built environments appear to influence behaviours relevant to weight status in young children, the magnitudes of these associations are generally modest and vary across contexts (Miller et al., 2014; Mizen et al., 2018; Mayor, 2014; Daniels et al., 2021a; Datar et al., 2013)

Other environmental factors such as housing type, garden size, area-level deprivation, and rurality also play an important role. Schalkwijk and colleagues (Schalkwijk et al., 2018b) reported that access to private gardens and greater neighbourhood greenness were positively associated with healthier weight outcomes in children aged 0-7 years living in England, highlighting the importance of unstructured outdoor play environments in early childhood (Maitland et al., 2013). In contrast, high-density housing and neighbourhood deprivation have been associated with higher stress levels and limited access to health-promoting resources, such as safe play spaces and healthy food options, which may contribute to increased risk of overweight and obesity (Singh et al., 2010). Rural environments, while often offering more open space, may lack walkable infrastructure or access to healthy food outlets, presenting a different set of challenges (Guseman et al., 2022; Kegler et al., 2009). Together, these findings suggest that multiple aspects of the residential environment interact to shape opportunities for physical activity, play, and dietary behaviours in young children.

Despite the evidence, major gaps remain. Few studies have examined how multifaceted neighbourhood environments collectively influence early childhood weight status, and most rely on small sample sizes or single-domain measures. Reviews have highlighted the need for more integrated approaches that capture the complexity of built environments and their combined influence on health (Van Der Horst et al., 2007; Sallis et al., 2012; Giles-Corti et al., 2016). The concept of an environmental phenotype – the combination of physical, social, and infrastructural characteristics of a neighbourhood that may shape health – offers an opportunity to fill this evidence gap.

Using high-resolution geographic information systems (GIS) data linked to child weight data from a national measurement programme, this study classifies residential phenotypes and examines their associations with weight in children aged 4-5 years living in Wales, within the United Kingdom. By using latent class analysis to identify environmental profiles, we move beyond single-exposure approaches to capture the broader configuration of neighbourhood characteristics experienced by children. In doing so, we provide a more holistic understanding of how combinations of environmental features relate to early childhood weight outcomes.

To our knowledge, this is the first population-level study to link multifaceted environmental data with early childhood weight data in this way, providing novel insights for research, policy, and urban planning. We hypothesise that children who live in health-promoting neighbourhood phenotypes are more likely to have a healthy weight status at age four and five, compared to children living in obesogenic phenotypes.

## 2 Methods

### 2.1 Data and measures

This observational study used routinely collected administrative data held in the Secure Anonymised Information Linkage (SAIL) Databank, hosted at Swansea University (Lyons et al., 2009; Jones et al., 2014, 2019). The SAIL Databank is an internationally recognised trusted research environment that provides access to rich, longitudinal health, socioeconomic and environmental datasets. Personally identifiable information is removed and replaced with unique Anonymised Linkage Fields (ALFs), enabling secure individual-level record linkage across multiple data sources. A corresponding Residential ALF (RALF) is also used to link individuals to their place of residence, allowing for linkage of both licensed and opensource environmental data to examine the characteristics of an individual's local environment. Full details of anonymisation and linkage methodology are published elsewhere (Lyons et al., 2009; Rodgers et al., 2009, 2012). Within this framework, multiple datasets were linked to characterise the residential locations of children and surrounding environmental features. These included the Child Measurement Programme (CMP) for measured child height and weight outcomes, the Welsh Demographic Service Dataset (WDSD) for residential location and demographic information, and a range of geospatial datasets to derive environmental characteristics surrounding each child's home.

The CMP for Wales provides robust public health surveillance data on the weight status of over 90% of children in state-maintained schools, using standardised height and weight measurements to estimate levels of overweight and obesity (The Child Measurement Programme for Wales Publication details Title, 2017). This study included children aged 4-5 with a measurement recorded in the CMP between 2012/2013 and 2018/19. Place of residence was captured for each child at the time of measurement. Children with missing identifiers or residential information were excluded.

### 2.2 Environmental phenotypes

Environmental variables were selected based on their potential relevance to young children's daily activity spaces and behaviours, including opportunities for outdoor play, access to recreational spaces, and exposures to food environments. These variables were also selected based on their potential to influence healthy weight outcomes, either as an environmental insult (e.g. high availability of hot food takeaways) or an environmental benefit (e.g. good access to green space). These data were combined to characterise the overall pattern of environmental features surrounding each home in Wales, which we refer to as an “environmental phenotype”. By this, we mean the combination of environmental characteristics within an area, rather than any single attribute considered in isolation.

The environmental data were derived at area level or neighbourhood level dependent on data availability. Neighbourhood was defined as the area within 900m of each child's residence, which was the closest to a 10-min walk we could achieve with the available data (Yang et al., 2021). A child-centred buffer was used to capture the local environment surrounding each child's residence and reflects the area that families are likely to access in their daily activities. As a result, neighbourhood areas may overlap for children living in close proximity.

### 2.3 Area level characteristics

Area-level data were used for variables that were only available at administrative geographic scales. The small-area geographic unit (Census geographies) for each residence, which corresponds to the Lower Layer Super Output Area (LSOA) in Wales, was extracted from the WDSD. LSOAs are standard administrative units with populations of approximately 1500 residents, providing consistent spatial scale for area-level variables. These were linked to the 2019 Welsh Index of Multiple Deprivation (WIMD) to obtain measures of socioeconomic status (deprivation). The WIMD is a composite index that combines indicators across multiple domains, including income, employment, health, education, housing, access to services, and the physical environment. Overall WIMD scores were assigned as quintiles, where 1 = most deprived and 5 = least deprived.

The ONS urban rural classification (Office for National Statistics, 2013) was also linked to residences, to investigate whether there were any differences in urban morphologies, and was categorised as: urban; urban in a sparse setting; rural town; rural town in a sparse setting; rural village; and rural village in a sparse setting. Urban areas are typically defined as settlements with populations of 10,000 or more, while rural areas include smaller towns, villages, and dispersed settlements. “Sparse settings” refer to areas with relatively low surrounding population density and greater geographic isolation compared with similar settlement types in less sparse regions.

### 2.4 Home and neighbourhood characteristics

Characteristics of the built environment were derived from one time point for the whole study period as previous research indicates that the built environment does not change considerably over time (Robinson et al., 2024; Hirsch et al., 2016a). Food outlets and greenspace data were derived from 2014 to 2017 respectively to align as closely as possible with the study mid-point and linked with children's residence at the time of CMP measurement.

Information on all points of interest (i.e., food outlets and greenspaces) within the neighbourhood were captured. Distance was categorised as hyper-local (0-300m), local (301-600m) or neighbourhood (601-900m).

#### 2.4.1 Greenspaces

Greenspace data were sourced from the 2017 Ordnance Survey (OS) Open Greenspaces dataset (OS open greenspace data), which depicts the location of publicly accessible areas such as parks and sports facilities. Access to green space is particularly important for young children as they may support outdoor play and physical activity. We defined greenspace as play space, playing field, public park or garden, tennis court, and other sports facility. Play spaces located within schools or paid-for tourist attractions were not included. Distance was calculated in metres from each residence to greenspace access point (vehicle or pedestrian) within 900m, with each greenspace counted only once regardless of multiple access points. The total number of greenspaces within 900m of each residence was used to capture overall exposure to outdoor environments. In addition, counts of specific greenspace types were included as separate variables to reflect potential differences in their use and relevance for young children.

Size of the nearest greenspace type was recorded in m^2^. Where there were multiple greenspaces of the same type located at equal distance, the largest greenspace was allocated. Greenspace size was categorised into ‘small’, ‘medium’, ‘large’, and ‘none’ based on the distribution of values for each individual greenspace type. Thresholds were derived from the observed range of values for each variable to create approximately even groupings. This ensured that the categorisation reflected the relative magnitude for each specific type of greenspace. These groupings were chosen to facilitate interpretation and support model convergence in the latent class analysis.

#### 2.4.2 Food outlets

The density of food outlets may influence dietary environments and exposure to energy-dense foods. Food outlet locations were derived from the 2014 OS Points of interest dataset and were included as a total number of food outlets within 900m of each residence and separate counts of specific outlet types. These included bakeries, cafes, confectioners (sweet/candy shop), convenience stores, hot food takeaways (including fish and chip shops), restaurants, and supermarkets. The total outlet measure was used to capture overall exposure to the local food environment, while individual outlet types were included to reflect potentially differing influences on dietary behaviours.

#### 2.4.3 House type

House type was included as it reflects housing density and the availability of private outdoor space, which may influence opportunities for outdoor play and physical activity among young children. House type was derived from Address Base Premium (AddressBase premium data products OS), which provides the location and attributes of residential and business addresses in the UK. The categories used were detached, semi-detached, self-contained flat, terraced, ‘other’, and ‘missing’ for all residences where house type was unavailable (11.1% of dwellings).

#### 2.4.4 Garden size

Access to outdoor space may provide safe spaces for outdoor play within the home environment. Garden polygons were extracted from OS MasterMap (OS MasterMap topography layer), which offers detailed vector mapping of land parcels, building footprints and other topographic features. Each garden polygon was assigned to the corresponding building using its Topographic Identifier (TOID), supplemented with land registry data to ensure accurate linkage between gardens and residential properties. Garden size was calculated in m^2^ and categorised into eight groups: 0-99.9 m^2^, 100-199.9 m^2^, 200-299.9 m^2^, 300-399.9 m^2^, 400-499.9 m^2^, 500 m^2^ +, no garden (for households with a garden size of zero) and ‘missing’ where no garden size data were available. Garden size data were unavailable for 9434 (5.6%) residential dwellings due to data linkage issues.

#### 2.4.5 Greenness

We operationalised Landsat 8 satellite imagery (30-m resolution) to calculate mean Enhanced Vegetation Index (EVI) value for all homes in Wales, which were then linked to children's addresses to capture a measure of residential greenness within 300m. EVI was derived from red, blue, and near-infrared (NIR) reflectance bands using the GRASS vegetation index tool in QGIS (QGIS manual. i). EVI work reported in this study was conducted as part of a wider longitudinal study (Garrett et al., 2023; Thompson et al., 2022; Mizen et al., 2019, 2024a) where a national level annual exposure variable was developed for 1.49 million households between 2008 and 2019. Full details of the EVI estimation methodology are described elsewhere (Thompson et al., 2022; Mizen et al., 2024a). EVI ranges from −1 to 1, where negative values generally correspond to water bodies, values near 0 indicate bare soil or built environments, and positive values indicate vegetation. Healthy vegetation is typically found in the 0·2 and 0·8 range (Huete et al., 2002; Mizen et al., 2024b).

#### 2.4.6 Walkability

Walkability was measured using the Welsh Active Living Environments Index, produced by Mah and colleagues (Mah et al., 2022) using data from the Wal-ALE database. Walkability is a metric of how conducive an area is to walking, based on various measures such as connectivity (e.g. footpaths, intersection density, points of interest), land use, safety, and street quality. Walkable areas are likely to promote active travel and access to local amenities.

The walkability index was calculated at the small-area geographic level and linked to addresses. Walkability was divided into quintiles to create an ordered scale from 1 (most walkable) to 5 (least walkable).

#### 2.4.7 Average distance to coast

The distance from the centroid of each small-area geographic unit to the nearest coastal location was applied to all addresses within that area to give an average distance to coast measure. Coastal environments may provide opportunities for outdoor recreation and physical activity, which promotes healthy behaviours in children.

#### 2.4.8 Nearest primary school

Proximity to primary schools may reflect neighbourhoods designed around family infrastructure and influence daily walking or travel patterns. Primary schools were identified from the Welsh Governments published school locations (Welsh Government). The network distance, using roads and footpaths, to the nearest primary school was calculated for each residence in metres and grouped into five categories: hyper-local, local, neighbourhood, >900m, and missing (0.2%).

### 2.5 Outcome measure – weight status

Children's Body Mass Index (BMI) was calculated from CMP height and weight data and categorised using UK1990 clinical reference standards into four groups: “underweight” (BMI <2nd centile), “healthy weight” (≥2nd to <91st), “overweight” (≥91st to <98th), and “obese” (≥98th), based on sex- and age-standardised z-scores. Z-scores beyond five standard deviations were excluded. For analysis, categories were simplified into a binary measure: 0 for healthy weight and 1 for unhealthy weight (overweight or obese), aligning with public health standards. This approach facilitates clearer interpretation of results for policy and practice audiences. Children categorised as underweight were excluded due to low numbers.

### 2.6 Statistical analysis

Latent Class Analysis (LCA) was used to identify the unknown (latent) groups of childhood neighbourhoods. Analysis was performed in STATA MP v18 to group households into distinct environmental phenotypes based on similarities across the 35 area-level, home and neighbourhood variables. For the points of interest variables, all counts that occurred in less than 1% of the sample were grouped together into an upper category to ensure sufficient cell sizes and to support model convergence during analysis.

The LCA method involves a number of steps: **1) specification of different models across a range of a number of groups and compare model fit.** LCA models with three classes through to nine were examined with model selection based on a combination of statistical fit indices (log-likelihood, AIC and BIC), entropy values, and consideration of class interpretability and plausibility. Models with lower information criterion indicate better fit and were plotted on a graph to identify points of diminishing return. In line with best practice, class size was also considered. A minimum threshold of 10% was applied to avoid identifying small, potentially unstable classes and to ensure the resulting phenotypes were meaningful at a population level. **2) select the model based on the optimal solution.** Emerging classes from the selected model were evaluated within the study group to determine whether their characteristics were both qualitatively and quantitively distinct and meaningful in the context of children's living environments, with phenotype labels assigned accordingly. Environmental phenotype labels were derived for each class based on a qualitative review of the characteristics and reflect the dominant or distinguishing features of each profile. **3) Assign each household to a distinct group.** Class assignment for each residence was based on the latent class with the highest posterior probability score generated by the LCA model.

To visually show the distribution of classes across Wales, the percentage of households belonging to each class was calculated for every small-area unit. Each unit was then assigned to the class with the highest proportion of residences. Boundary shapefiles for Wales (2011) were obtained from the Office for National statistics Open Geography Portal. A thematic map was generated using the *tmap* package in R (tmap, 2018).

#### 2.6.1 Logistic regression

Associations between environmental phenotype and weight status were analysed using logistic regression. Each child was linked to an environmental phenotype based on their place of residence at the time of their BMI measurement. We adjusted for age and sex to enable evaluation of differences between these groups. We assumed statistical independence between all children, including those registered to the same address. R^2^ value is reported as an indication of overall variation explained by neighbourhood phenotype membership.

## 3 Results

The environmental phenotypes were generated based on the environmental characteristics of 167,789 unique houses in which children resided. Full details of the physical environmental characteristics can be found in appendix 1.

### 3.1 Latent Class Analysis

The AIC and BIC model statistics for three through eight classes improved with increasing number of classes, however, the rate of improvement was minimal beyond five classes. This suggests that adding classes beyond this point would yield minimal additional explanatory value and may overfit the data. Additionally, models with six or more classes included at least one class with less than 10% of the sample. The model with nine classes failed to converge and deemed to be a poor fit. The model with five classes was therefore selected as the best fitting model. The five environmental phenotypes were subsequently labelled: *Rural, spacious and isolated*, *Suburban*, *Deprived and underserved*, *Deprived and well-served*, and *Dense, coastal and well-connected*.

The *Rural, spacious and isolated* phenotype represented 14% (24,266) of residences and contained predominantly detached homes, the largest gardens and the highest proportion of neighbourhood vegetation. This profile had very limited access to designated greenspaces, food outlets and primary schools. The *Suburban* phenotype represented 17% (29,324) of residences and was characterised most notably by the presence of semi-detached housing with low levels of deprivation. It also had good access to the coast and to greenspaces, and very low counts of food outlets. The *Deprived and underserved* phenotype represented 23% (39,227) of residences and was predominantly urban with terraced housing in deprived areas. Households in this group also had limited access to greenspace overall but had higher counts of children's play spaces in the hyper-local area. The *Deprived and well-served* phenotype represented 32% (53,210) of residences and was similar to profile three except having better access to primary schools, and higher counts of food outlets and greenspaces. The final phenotype, *Dense, coastal and well-connected* represented 13% (21,762) of residences and had predominantly terraced housing in deprived, urban areas with the smallest gardens. This group had higher counts of hot food takeaways in their local neighbourhood compared with households in other phenotypes. Households were also closest to the coast, primary schools and had better access to the largest greenspaces. Summary characteristics of the five environmental phenotypes can be seen in Table 1 and a full breakdown in Appendix 1.

**Table 1:** Summary characteristics of environmental phenotypes.

Phenotype	Socioeconomic deprivation	Greenspace	Food environment	Urbanicity	Housing/gardens
**Rural, spacious and isolated**	Very low	Very low access	Very low	Rural/sparse	Large gardens, detached housing
**Suburban**	Low	Moderate	Low	Mixed suburban	Medium gardens, semi-detached
**Deprived and underserved**	High	Limited (few large spaces, some play spaces)	Moderate	Urban	Medium gardens, more terraced housing
**Deprived and well-served**	High	Good access (larger parks, facilities)	High	Urban	Medium gardens, dense housing
**Dense, coastal and well-connected**	High	Very high access; greater access to the largest greenspaces	Very high (especially hot food takeaways)	Highly urban/closest to the coast	Smallest gardens, high-density terraced housing

**Fig. 1:**
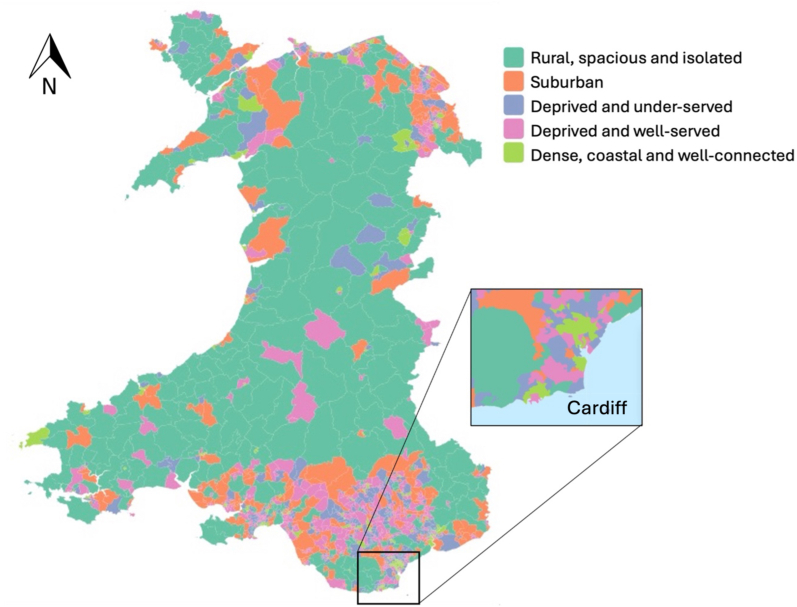
Phenotype distribution by small-area geography across Wales, United Kingdom.

**Fig. 2:**
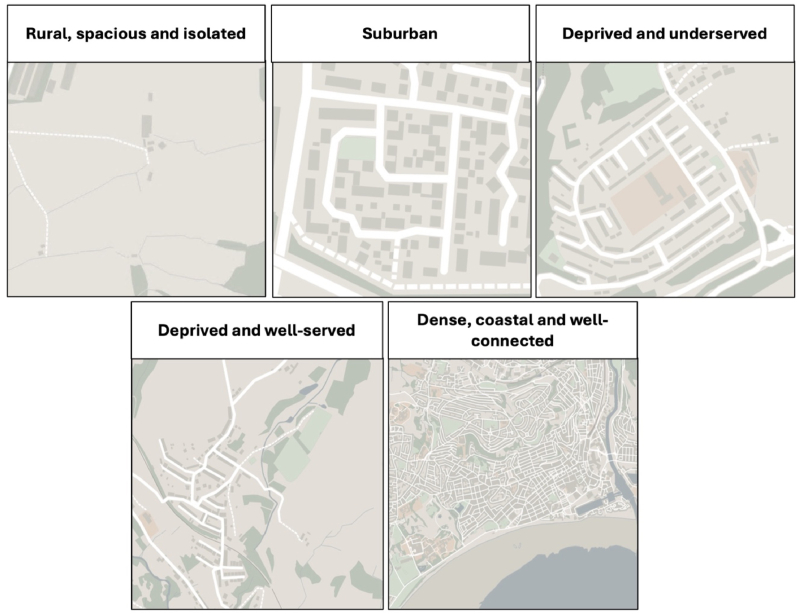
Representative example of how each environmental phenotype would look geographically.

Fig. 1 illustrates the geographic distribution of phenotypes across Wales to highlight the spatial variation in neighbourhood environments experienced by children. An inset map of the capital city, Cardiff, is also included to provide greater detail of the urbanised metropolitan area. Fig. 2 provides representative examples of the characteristics associated with each phenotype, illustrating how combinations of features such as housing type, greenspace availability, and local amenities differ across neighbourhood types.

### 3.2 Association between residential environment and weight status

The study included 214,947 children aged 4-5 years, of whom 18% were classified as having an unhealthy weight (Table 2). The sample was evenly distributed by gender (51% boys, 49% girls), and most children were aged 5 years (58%). Almost one third (32%) of children lived in the phenotype *Deprived and well served*. Overall, the distribution of weight status was broadly similar across demographic groups.

**Table 2:** Children in the Child Measurement Programme (CMP) by weight category (n = 214,947).

	N (%)		Healthy weight (%)		Unhealthy weight (%)	
N	214,947	(100)	176,301	(82)	38,646	(18)

**Gender**						
Boys	109,688	(51)	89,548	(82)	20,140	(18)
Girls	105,259	(49)	86,753	(82)	18,506	(18)

**Age**						
4 years	91,312	(42)	74,563	(82)	16,749	(18)
5 years	123,635	(58)	101,738	(82)	21,897	(18)

**Environmental Phenotype**						
Rural, spacious and isolated	31,248	(15)	26,180	(84)	5068	(16)
Suburban	37,544	(17)	30,782	(82)	6762	(18)
Deprived and underserved	50,150	(23)	41,227	(82)	8923	(18)
Deprived and well-served	68,163	(32)	55,123	(81)	13,040	(19)
Dense, coastal and well-connected	27,842	(13)	22,989	(83)	4853	(17)

After adjusting for age and sex, children who lived in *Deprived and well-served* environments had the highest odds of being an unhealthy weight and were 9% (OR = 1·09; 95% CI: 1·06 – 1·13) more likely to be living with an unhealthy weight compared to children living in *Deprived and under-served* environments. Children living in *Rural, spacious and isolated* environments, had the lowest odds of being an unhealthy weight. These children were 11% (OR = 0·89; 95% CI: 0·86 – 0·93) less likely to have an unhealthy weight compared to children living in class three.

As shown in Table 3, girls were less likely to be an unhealthy weight compared to boys (OR = 0.95; 95% CI: 0.93-0.97) and children aged five had a lower odds of being an unhealthy weight compared to children aged four (OR = 0.93; 95% CI: 0.90-0.96).

**Table 3:** Logistic regression estimates for the association between environmental phenotypes and children's weight category (healthy weight vs. unhealthy weight).

	Odds ratio	95% Confidence Interval
**Profile**		
Rural, spacious and isolated	0.89	0.86 - 0.93
Suburban	1.02	0.98 - 1.05
Deprived and under-served (*reference*)	-	-
Deprived and well-served	1.09	1.06 - 1.13
Dense, coastal and well-connected	0.98	0.94 - 1.01
**Age**		
4 years (*reference*)	-	-
5 years	0.93	0.90 - 0.96
**Sex**		
Boys (*reference*)	-	-
Girls	0.95	0.93 - 0.97

*R*^*2*^*= 0.0009*		

## 4 Discussion

This study is the first to use population-level health and multi-faceted environmental exposure data to examine associations between environmental phenotypes and early childhood weight status. Consistent with previous research (Schalkwijk et al., 2018b; Jia et al., 2021; Van Der Zwaard et al., 2018), findings suggest that environments associated with healthy weight at age four and five were characterised by rurality, low fast-food density and opportunities for both structured and unstructured play in private gardens. In contrast, children in more urban, deprived settings with high fast-food density benefitted from protective features such as accessible greenspace and coastal areas, highlighting the importance of publicly available play opportunities when private outdoor space is limited.

The *Deprived and underserved* and *Deprived and well-served* phenotypes showed similar levels of deprivation, however, the *Deprived and well-served* group had greater access to food outlets and greenspaces overall, whereas the *Deprived and underserved* group had fewer neighbourhood-level greenspaces but a higher density of hyper-local play spaces alongside fewer hot food takeaways. While total food outlet density captures overall exposure to the local food environment, it is important to recognise that this comprises a heterogeneous mix of outlet types, including both potentially health-promoting (e.g. supermarkets) and less healthy options (e.g. hot food takeaways). In this study, both total outlet density and counts of specific outlet types were included, allowing us to capture variation in overall exposure as well as differences in exposure to specific food outlet types. Consistent with previous research (Daniels et al., 2021a; Welsh Government; tmap, 2018; Jia et al., 2021; Van Der Zwaard et al., 2018), results suggest that even in highly deprived settings where there is often an accumulation of obesogenic features (Jenkin G et al., 2015), the availability of accessible, child-focused play spaces combined with reduced exposure to food outlets may provide important protective factors for maintaining healthy weight in early childhood. This supports the growing evidence that it is not single exposures, but combinations of built environment features that collectively shape health outcomes. Policy interventions which target areas based on deprivation in addition to other phenotype characteristics such as increased play spaces and fewer food outlets may be more effective.

By applying a latent class approach, we were able to define distinct environmental phenotypes rather than assessing individual environmental features in isolation. This multidimensional perspective offers a more holistic understanding of how contextual factors interact to influence childhood weight status. Although existing evidence indicates that associations between environmental features and childhood weight status are generally weak, prior studies have relied on narrow exposure measures (Poole and Moon, 2017) or area-level proxies (Beynon et al., 2021). While the observed differences in unhealthy weight prevalence across environmental phenotypes were relatively modest (16%–19%), this is broadly consistent with findings from other population-level studies examining neighbourhood influences on early childhood weight (Daniels et al., 2021a; Jia et al., 2021). These findings can also be understood within ecological frameworks of child development, such as Bronfenbrenner's (Urie, 1979) ecological systems theory, which emphasises the interacting influence of multiple environments including family, neighbourhoods, and wider policy contexts. Within this framework, neighbourhood characteristics represent one layer of influence that may shape opportunities for physical activity, access to food, and outdoor play. The modest differences observed across phenotypes are consistent with the idea that childhood weight is shaped by multiple, interacting systems rather than any single environmental factor. Nevertheless, understanding environmental contributions remains important for identifying population-level interventions and planning health-promoting neighbourhoods. Using a phenotype-based approach allows us to capture the complexity of children's lived environments and how these configurations promote or inhibit healthy behaviours.

### 4.1 Strengths and limitations

A key strength of this study is the linkage of a national dataset (CMP) with objectively measured environmental exposures via the SAIL Databank, enabling the first population-level classification of home environment phenotypes across Wales. This data-driven approach allowed us to integrate multiple exposures and identify meaningful patterns associated with weight status.

The child measurement programme is conducted in state-maintained schools and therefore does not include children attending private schools or those who are homeschooled. While coverage is high, this may result in a small proportion of the child population not being represented. We also acknowledge the limitations of using BMI as an indicator of overweight and obesity. Whilst it is a widely used and practical measure of weight status in children, it does not distinguish between body fat and lean mass and may not fully capture differences in body composition across subpopulations.

Other limitations include reliance on proximity-based measures, which may not reflect utilisation or the quality of spaces accessible to children. Further, young children's behaviours are dictated by parental preferences which is also true for dietary behaviours of the children and household. This study is observational in nature, meaning that findings should be interpreted as associations rather than causal effects. We were unable to account for potential sources of endogeneity, including residential selection into neighbourhood phenotypes. Although we adjusted for key sociodemographic factors, residual confounding and unmeasured selection effects may remain. While approaches such as comparing children within smaller geographic areas may help to address unobserved heterogeneity, the area-based nature of the environmental phenotypes limits the feasibility of such analyses within the current framework, as this would substantially reduce variability in exposures. It should also be noted that deprivation was included as an indicator in the latent class analysis, meaning that some environmental characteristics may still be patterned by socioeconomic context.

Points of interest and greenspace data were derived from a single time point to align with the study mid-point and as a result, temporal changes to the built environment before and after this period were not captured. Additionally, home environment phenotypes were assigned based on place of residence at the time of CMP measurement and do not reflect longitudinal exposures. Despite these limitations, research indicates that the built environment does not change considerably over time (Hirsch et al., 2016b) and that families in Wales tend to remain in similar areas in terms of environmental characteristics (Davies et al., 2024). While differences in characteristics are likely to be minimal, we acknowledge that employing a longitudinal design would allow changes in exposures to be measured over time and better linked to child health trajectories.

We assumed independence between observations as a reliable family-level identifier was not available to account for clustering of children within households. As a result, children living in the same household either concurrently or at different time points, were assigned to the same latent class and may share behavioural similarities that influence weight. Future work could apply more advanced methodology to account for within-household clustering and shared behaviours.

Although the logistic regression model demonstrated statistically significant associations between built environment phenotypes and childhood weight status, the overall explanatory power was limited (as indicated by a low pseudo-R^2^ value). This is not unexpected, as childhood obesity is a multifactorial outcome influenced by a wide range of determinants; however, the results demonstrate that environmental phenotypes play an important role in shaping differences in population level childhood weight status.

### 4.2 Directions for future research

Future research should integrate behavioural and lifestyle data, such as information captured by supermarket loyalty schemes and digital devices, to better quantify the use of environmental features. Analyses should also be extended to include older children as they gain greater independent mobility, and additional exposures should be considered, such as access to play spaces in schools. Future work could also examine how mobility between environmental phenotypes influences childhood weight status. While our study focussed on healthy weight of children aged 4-5, our method provides a categorisation of environmental phenotypes that can be used to investigate associations with any number of health outcomes.

Our study demonstrates the value of defining environmental phenotypes for evaluating the role of the place around children's homes on their physical health and highlights the importance of conducting individual-level data linkages. Given the public health challenge of childhood obesity, identifying modifiable risk factors is crucial. By uncovering the neighbourhood characteristics associated with childhood weight status, this research provides policy makers with an important evidence-base to design targeted, place-based interventions that can help transform neighbourhoods into environments that support healthier weight trajectories for children.

#### Funding

This work is part of the Built Environment and Child Health in Wales and Australia (BEACHES) project which is a joint initiative between Telethon Kids Institute, University of Western Australia and Swansea University. The BEACHES Project is funded by the UKRI-NHMRC Built Environment Prevention Research Scheme (grant number GNT1192764 and MR/T039329/1). Administrative Data Research (ADR) Wales also supported this research, which forms part of the ADR UK investment that unites research expertise from Swansea University Medical School and WISERD (Wales Institute of Social and Economic Research and Data) at Cardiff University with analysts from Welsh Government. ADR UK is funded by the Economic and Social Research Council (ESRC), part of UK Research and Innovation. Hayley Christian is supported by an Australian National Heart Foundation Future Leader Fellowship (102549) and partially supported by the Australian Government through the Australian Research Council's Centre of Excellence for Children and Families over the Life Course (Project ID CE200100025).

## CRediT authorship contribution statement

**Jo Davies:** Conceptualization, Formal analysis, Investigation, Methodology, Project administration, Writing – original draft, Writing – review & editing. **Rowena Bailey:** Conceptualization, Formal analysis, Investigation, Methodology, Validation, Writing – review & editing. **Rebecca Pedrick-Case:** Conceptualization, Data curation, Methodology, Validation, Writing – review & editing. **Gareth Stratton:** Conceptualization, Funding acquisition, Methodology, Project administration, Supervision, Writing – review & editing. **Theodora Pouliou:** Conceptualization, Investigation, Writing – review & editing. **Amy Mizen:** Writing – review & editing. **Hayley Christian:** Conceptualization, Methodology, Writing – review & editing. **Bryan Boruff:** Conceptualization, Writing – review & editing. **Ronan A. Lyons:** Conceptualization, Supervision, Writing – review & editing. **Rich Fry:** Conceptualization, Funding acquisition, Investigation, Methodology, Supervision, Writing – original draft, Writing – review & editing. **Lucy J. Griffiths:** Conceptualization, Funding acquisition, Investigation, Methodology, Project administration, Supervision, Writing – original draft, Writing – review & editing.

## Figures and Tables

**Table:** 

Profile name	Rural, spacious and isolated				Suburban				Deprived and underserved				Deprived and well-served				Dense, coastal and well-connected			
Number households (%)	24,266		14.5		29,324		17.5		39,227		23.4		53,210		31.7		21,762		13.0	
Characteristic	Median	(IQR)	Range		Median	(IQR)	Range		Median	(IQR)	Range		Median	(IQR)	Range		Median	(IQR)	Range	
Parks & gardens	0	(0)	0	13	0	(0)	0	15	0	(2)	0	15	0	(2)	0	15	4	(7)	0	15
Playing fields	0	(0)	0	0	2	(3)	1	13	0	(0)	0	0	3	(4)	1	13	1	(4)	0	13
Play spaces	0	(1)	0	7	1	(2)	0	10	2	(2)	0	10	2	(2)	0	10	2	(3)	0	10
Tennis courts	0	(0)	0	0	0	(0)	0	0	0	(0)	0	1	0	(0)	0	1	0	(0)	0	1
Other sports facilities	0	(0)	0	10	0	(0)	0	13	0	(1)	0	13	0	(2)	0	13	1	(3)	0	13
Total greenspaces	0	(1)	0	13	4	(3)	1	25	3	(4)	0	27	9	(6)	1	27	12	(9)	0	27
Bakeries	0	(0)	0	2	0	(0)	0	4	0	(0)	0	6	0	(1)	0	6	2	(2)	0	6
Cafes	0	(0)	0	4	0	(0)	0	6	0	(1)	0	18	0	(1)	0	18	5	(7)	0	18
Confectioners	0	(0)	0	1	0	(0)	0	1	0	(0)	0	3	0	(0)	0	3	1	(1)	0	3
Convenience stores	0	(0)	0	8	1	(2)	0	7	2	(2)	0	19	2	(2)	0	19	6	(6)	0	19
Fast food outlets	0	(0)	0	10	0	(1)	0	10	1	(3)	0	28	2	(4)	0	28	12	(11)	0	28
Restaurants	0	(0)	0	10	0	(0)	0	9	0	(1)	0	22	0	(1)	0	22	4	(8)	0	22
Supermarkets	0	(0)	0	3	0	(0)	0	3	0	(1)	0	4	0	(1)	0	4	1	(2)	0	4
Total food outlets	0	(0)	0	17	1	(3)	0	25	4	(5)	0	82	7	(7)	1	82	32	(29)	3	82
EVI	0.4	(0.2)	0.2	0.8	0.3	(0.1)	0	0.7	0.3	(0.2)	0	0.7	0.3	(0.1)	0	0.7	0.2	(0.2)	0	0.6
	**Frequency**	**%**			**Frequency**	**%**			**Frequency**	**%**			**Frequency**	**%**			**Frequency**	**%**		
Size nearest Park or Garden																				
No park or garden	23,456	96.7	-	-	26,998	92.1	-	-	26,102	66.5	-	-	34,421	64.7	-	-	4396	20.2	-	-
Small	104	0.4	-	-	765	2.6	-	-	2722	6.9	-	-	5661	10.6	-	-	4922	22.6	-	-
Medium	179	0.7	-	-	663	2.3	-	-	4630	11.8	-	-	6203	11.7	-	-	7193	33.1	-	-
Large	527	2.2	-	-	898	3.1	-	-	5773	14.7	-	-	6925	13.0	-	-	5251	24.1	-	-
Size nearest Playing field																				
No playing field	24,266	100.0	-	-	0	0.0	-	-	39,227	100.0	-	-	0	0.0	-	-	8787	40.4	-	-
Small	0	0.0	-	-	12,230	41.7	-	-	0	0.0	-	-	13,184	24.8	-	-	4116	18.9	-	-
Medium	0	0.0	-	-	9044	30.8	-	-	0	0.0	-	-	18,987	35.7	-	-	3893	17.9	-	-
Large	0	0.0	-	-	8050	27.5	-	-	0	0.0	-	-	21,039	39.5	-	-	4966	22.8	-	-
Size nearest play space																				
No play space	17,665	72.8	-	-	9019	30.8	-	-	7461	19.0	-	-	5156	9.7	-	-	3728	17.1	-	-
Small	2953	12.2	-	-	7969	27.2	-	-	12,438	31.7	-	-	16,765	31.5	-	-	4037	18.6	-	-
Medium	2040	8.4	-	-	7097	24.2	-	-	9359	23.9	-	-	15,961	30.0	-	-	6741	31.0	-	-
Large	1608	6.6	-	-	5239	17.9	-	-	9969	25.4	-	-	15,328	28.8	-	-	7256	33.3	-	-
Size nearest tennis court																				
No tennis court	24,266	100.0	-	-	29,324	100.0	-	-	38,997	99.4	-	-	52,807	99.2	-	-	20,707	95.2	-	-
Small	0	0.0	-	-	0	0.0	-	-	46	0.1	-	-	209	0.4	-	-	237	1.1	-	-
Medium	0	0.0	-	-	0	0.0	-	-	13	0.0	-	-	153	0.3	-	-	589	2.7	-	-
Large	0	0.0	-	-	0	0.0	-	-	171	0.4	-	-	41	0.1	-	-	229	1.1	-	-
Size nearest other sports facility																				
No other sports facility	23,900	98.5	-	-	27,537	93.9	-	-	26,849	68.4	-	-	31,184	58.6	-	-	9580	44.0	-	-
Small	146	0.6	-	-	817	2.8	-	-	4770	12.2	-	-	7723	14.5	-	-	4728	21.7	-	-
Medium	128	0.5	-	-	425	1.4	-	-	3375	8.6	-	-	6457	12.1	-	-	3001	13.8	-	-
Large	92	0.4	-	-	545	1.9	-	-	4233	10.8	-	-	7846	14.7	-	-	4453	20.5	-	-
Garden size (m^2^)																				
No Garden	1356	5.6	-	-	911	3.1	-	-	704	1.8	-	-	746	1.4	-	-	550	2.5	-	-
0-99.9	2198	9.1	-	-	4672	15.9	-	-	9352	23.8	-	-	13,970	26.3	-	-	11,134	51.2	-	-
100-199.9	5016	20.7	-	-	9679	33.0	-	-	13,558	34.6	-	-	18,030	33.9	-	-	4375	20.1	-	-
200-299.9	3961	16.3	-	-	6385	21.8	-	-	7813	19.9	-	-	10,704	20.1	-	-	1922	8.8	-	-
300-399.9	2179	9.0	-	-	2701	9.2	-	-	2861	7.3	-	-	3672	6.9	-	-	705	3.2	-	-
400-499.9	1484	6.1	-	-	1275	4.3	-	-	1180	3.0	-	-	1357	2.6	-	-	305	1.4	-	-
500+	6823	28.1	-	-	2507	8.5	-	-	1944	5.0	-	-	1859	3.5	-	-	463	2.1	-	-
Missing	1249	5.1	-	-	1194	4.1	-	-	1815	4.6	-	-	2872	5.4	-	-	2308	10.6	-	-
Nearest primary school																				
Hyper-local	1406	5.8	-	-	3323	11.3	-	-	4527	11.5	-	-	9038	17.0	-	-	4877	22.4	-	-
Local	2507	10.3	-	-	7546	25.7	-	-	11,140	28.4	-	-	19,083	35.9	-	-	9071	41.7	-	-
Neighbourhood	2880	11.9	-	-	8072	27.5	-	-	10,520	26.8	-	-	15,345	28.8	-	-	4903	22.5	-	-
>900m	17,445	71.9	-	-	10,344	35.3	-	-	12,981	33.1	-	-	9655	18.1	-	-	2814	12.9	-	-
Missing	28	0.1	-	-	39	0.1	-	-	59	0.2	-	-	89	0.2	-	-	97	0.4	-	-
Average distance to coast (miles)																				
<1	356	1.5	-	-	323	1.1	-	-	891	2.3	-	-	1080	2.0	-	-	1392	6.4	-	-
1	1961	8.1	-	-	2996	10.2	-	-	4442	11.3	-	-	5652	10.6	-	-	4074	18.7	-	-
2	1495	6.2	-	-	1840	6.3	-	-	3366	8.6	-	-	3058	5.7	-	-	3408	15.7	-	-
3	1333	5.5	-	-	2360	8.0	-	-	2611	6.7	-	-	4559	8.6	-	-	1630	7.5	-	-
4	2101	8.7	-	-	1793	6.1	-	-	4146	10.6	-	-	3785	7.1	-	-	1888	8.7	-	-
5	1035	4.3	-	-	1952	6.7	-	-	2400	6.1	-	-	3159	5.9	-	-	2026	9.3	-	-
6	2017	8.3	-	-	2274	7.8	-	-	1964	5.0	-	-	3685	6.9	-	-	651	3.0	-	-
7-10	4050	16.7	-	-	4482	15.3	-	-	4591	11.7	-	-	5772	10.8	-	-	1606	7.4	-	-
11-14	3012	12.4	-	-	3737	12.7	-	-	3574	9.1	-	-	6942	13.0	-	-	1314	6.0	-	-
15+	6906	28.5	-	-	7567	25.8	-	-	11,242	28.7	-	-	15,518	29.2	-	-	3773	17.3	-	-
Walkability index																				
1 - Most Walkable	15,701	64.7	-	-	12,378	42.21	-	-	7405	18.88	-	-	8361	15.71	-	-	1111	5.11	-	-
2	3057	12.6	-	-	6437	21.95	-	-	11,661	29.73	-	-	16,847	31.66	-	-	7003	32.18	-	-
3	370	1.52	-	-	1696	5.78	-	-	6109	15.57	-	-	9859	18.53	-	-	6453	29.65	-	-
4	5138	21.17	-	-	8803	30.02	-	-	13,927	35.5	-	-	17,666	33.2	-	-	3490	16.04	-	-
5 - Least walkable	-	-	-	-	10	0.03	-	-	125	0.32	-	-	477	0.9	-	-	3705	17	-	-
House type																				
Detached	11,516	47.5	-	-	7262	24.8	-	-	7248	18.5	-	-	5181	9.7	-	-	1267	5.8	-	-
Self-Contained Flat	206	0.8	-	-	567	1.9	-	-	1083	2.8	-	-	1725	3.2	-	-	1562	7.2	-	-
Semi-Detached	5543	22.8	-	-	10,304	35.1	-	-	11,458	29.2	-	-	17,968	33.8	-	-	3552	16.3	-	-
Terraced	3733	15.4	-	-	8205	28.0	-	-	14,286	36.4	-	-	23,199	43.6	-	-	13,119	60.3	-	-
Other	84	0.3	-	-	20	0.1	-	-	18	0.0	-	-	13	0.0	-	-	68	0.3	-	-
Missing	3184	13.1	-	-	2966	10.1	-	-	5134	13.1	-	-	5124	9.6	-	-	2194	10.1	-	-
Urban Rural classification																				
City & town less sparse	9328	38.4	-	-	17,509	59.7	-	-	31,209	79.6	-	-	41,737	78.4	-	-	18,385	84.5	-	-
City & town sparse	271	1.1	-	-	803	2.7	-	-	477	1.2	-	-	770	1.4	-	-	660	3.0	-	-
Town & fringe less sparse	2904	12.0	-	-	5741	19.6	-	-	4561	11.6	-	-	8384	15.8	-	-	1298	6.0	-	-
Town & fringe sparse	751	3.1	-	-	682	2.3	-	-	1090	2.8	-	-	1763	3.3	-	-	1323	6.1	-	-
Village less sparse	4664	19.2	-	-	3047	10.4	-	-	809	2.1	-	-	274	0.5	-	-	14	0.1	-	-
Village sparse	6348	26.2	-	-	1542	5.3	-	-	1081	2.8	-	-	282	0.5	-	-	82	0.4	-	-
WIMD^a^																				
1 - Most deprived	1032	4.3	-	-	4548	15.5	-	-	10,968	28.0	-	-	18,366	34.5	-	-	7049	32.4	-	-
2	3124	12.9	-	-	6029	20.6	-	-	7887	20.1	-	-	12,705	23.9	-	-	5740	26.4	-	-
3	5873	24.2	-	-	5387	18.4	-	-	5691	14.5	-	-	9893	18.6	-	-	3986	18.3	-	-
4	7725	31.8	-	-	6795	23.2	-	-	5151	13.1	-	-	6862	12.9	-	-	3037	14.0	-	-
5 - Least deprived	6512	26.8	-	-	6565	22.4	-	-	9530	24.3	-	-	5384	10.1	-	-	1950	9.0	-	-
WIMD Income																				
1 - Most deprived	2555	10.5	-	-	4270	14.6	-	-	7992	20.4	-	-	10,759	20.2	-	-	8943	41.1	-	-
2	3720	15.3	-	-	5557	19.0	-	-	9024	23.0	-	-	11,488	21.6	-	-	4590	21.1	-	-
3	5049	20.8	-	-	5800	19.8	-	-	7936	20.2	-	-	11,561	21.7	-	-	3454	15.9	-	-
4	6611	27.2	-	-	6989	23.8	-	-	7368	18.8	-	-	9986	18.8	-	-	1892	8.7	-	-
5 - Least deprived	6331	26.1	-	-	6708	22.9	-	-	6907	17.6	-	-	9416	17.7	-	-	2883	13.2	-	-
WIMD Employment																				
1 - Most deprived	1161	4.8	-	-	4933	16.8	-	-	11,251	28.7	-	-	17,456	32.8	-	-	5678	26.1	-	-
2	2442	10.1	-	-	5961	20.3	-	-	7650	19.5	-	-	14,120	26.5	-	-	6302	29.0	-	-
3	4156	17.1	-	-	6189	21.1	-	-	6926	17.7	-	-	11,468	21.6	-	-	4766	21.9	-	-
4	7719	31.8	-	-	5880	20.1	-	-	6100	15.6	-	-	6308	11.9	-	-	3075	14.1	-	-
5 - Least deprived	8788	36.2	-	-	6361	21.7	-	-	7300	18.6	-	-	3858	7.3	-	-	1941	8.9	-	-
WIMD Health																				
1 - Most deprived	1228	5.1	-	-	5364	18.3	-	-	11,178	28.5	-	-	18,077	34.0	-	-	5971	27.4	-	-
2	2299	9.5	-	-	6198	21.1	-	-	8038	20.5	-	-	14,058	26.4	-	-	5676	26.1	-	-
3	3625	14.9	-	-	6382	21.8	-	-	7070	18.0	-	-	11,756	22.1	-	-	5195	23.9	-	-
4	8317	34.3	-	-	5867	20.0	-	-	6243	15.9	-	-	5968	11.2	-	-	2832	13.0	-	-
5 - Least deprived	8797	36.3	-	-	5513	18.8	-	-	6698	17.1	-	-	3351	6.3	-	-	2088	9.6	-	-
WIMD Education																				
1 - Most deprived	1225	5.0	-	-	5246	17.9	-	-	11,270	28.7	-	-	19,057	35.8	-	-	5042	23.2	-	-
2	2506	10.3	-	-	5891	20.1	-	-	8058	20.5	-	-	12,881	24.2	-	-	6257	28.8	-	-
3	4351	17.9	-	-	6500	22.2	-	-	6224	15.9	-	-	11,066	20.8	-	-	4560	21.0	-	-
4	8792	36.2	-	-	5515	18.8	-	-	6351	16.2	-	-	6132	11.5	-	-	3075	14.1	-	-
5 - Least deprived	7392	30.5	-	-	6172	21.0	-	-	7324	18.7	-	-	4074	7.7	-	-	2828	13.0	-	-
WIMD Access																				
1 - Most deprived	12,469	51.4	-	-	6181	21.1	-	-	5295	13.5	-	-	4600	8.6	-	-	1299	6.0	-	-
2	2824	11.6	-	-	7334	25.0	-	-	9090	23.2	-	-	13,286	25.0	-	-	3942	18.1	-	-
3	3174	13.1	-	-	6299	21.5	-	-	8046	20.5	-	-	13,323	25.0	-	-	4975	22.9	-	-
4	2927	12.1	-	-	5260	17.9	-	-	8426	21.5	-	-	11,478	21.6	-	-	5541	25.5	-	-
5 - Least deprived	2872	11.8	-	-	4250	14.5	-	-	8370	21.3	-	-	10,523	19.8	-	-	6005	27.6	-	-
WIMD Houing																				
1 - Most deprived	4692	19.3	-	-	2889	9.9	-	-	4425	11.3	-	-	8735	16.4	-	-	10,660	49.0	-	-
2	4134	17.0	-	-	4792	16.3	-	-	8285	21.1	-	-	13,806	25.9	-	-	4393	20.2	-	-
3	4302	17.7	-	-	6547	22.3	-	-	8431	21.5	-	-	13,002	24.4	-	-	3170	14.6	-	-
4	4414	18.2	-	-	7461	25.4	-	-	8032	20.5	-	-	12,081	22.7	-	-	2589	11.9	-	-
5 - Least deprived	6724	27.7	-	-	7635	26.0	-	-	10,054	25.6	-	-	5586	10.5	-	-	950	4.4	-	-
WIMD Community Safety																				
1 - Most deprived	1521	6.3	-	-	3184	10.9	-	-	8582	21.9	-	-	15,715	29.5	-	-	9006	41.4	-	-
2	1914	7.9	-	-	5863	20.0	-	-	8140	20.8	-	-	14,881	28.0	-	-	5534	25.4	-	-
3	3391	14.0	-	-	6319	21.5	-	-	7867	20.1	-	-	11,554	21.7	-	-	4393	20.2	-	-
4	6505	26.8	-	-	7291	24.9	-	-	8183	20.9	-	-	8002	15.0	-	-	2199	10.1	-	-
5 - Least deprived	10,935	45.1	-	-	6667	22.7	-	-	6455	16.5	-	-	3058	5.7	-	-	630	2.9	-	-
WIMD Physical Environment																				
1 - Most deprived	2555	10.5	-	-	4270	14.6	-	-	7992	20.4	-	-	10,759	20.2	-	-	8943	41.1	-	-
2	3720	15.3	-	-	5557	19.0	-	-	9024	23.0	-	-	11,488	21.6	-	-	4590	21.1	-	-
3	5049	20.8	-	-	5800	19.8	-	-	7936	20.2	-	-	11,561	21.7	-	-	3454	15.9	-	-
4	6611	27.2	-	-	6989	23.8	-	-	7368	18.8	-	-	9986	18.8	-	-	1892	8.7	-	-
5 - Least deprived	6331	26.1	-	-	6708	22.9	-	-	6907	17.6	-	-	9416	17.7	-	-	2883	13.2	-	-

a WIMD – Welsh Index of Multiple Deprivation (measure of small-area geography).

**Table:** 

Class	n	ll(null)	ll(model)	df	AIC	BIC
threeclass	167,789	.	1973838	107	3947890	3948963
fourclass	167,789	.	1926573	143	3853432	3854866
fiveclass	167,789	.	1857564	178	3715485	3717270
sixclass	167,789	.	1818534	215	3637498	3639654
sevenclass	167,789	.	1760539	251	3521581	3524098
eightclass	167,789	.	1739441	287	3479456	3482335

## Data Availability

The authors do not have permission to share data.

## Appendix 2

### Latent class analysis - model fit statistics

The model with nine classes failed to converge. The model with eight classes had the lowest AIC value, however the six, seven and eight class models all had classes that contained less than 10% of the sample. The five class model was selected as the best fitting model.

## References

[bib1] AddressBase premium data products OS n.d. https://www.ordnancesurvey.co.uk/products/addressbase-premium (accessed July 2, 2025).

[bib2] Beynon C., Pashayan N., Fisher E., Hargreaves D.S., Bailey L., Raine R. (2021). A cross-sectional study using the childhood measurement programme for Wales to examine population-level risk factors associated with childhood obesity. Public Health Nutr..

[bib3] 2011 census geographies - Office for national statistics n.d.https://www.ons.gov.uk/methodology/geography/ukgeographies/censusgeographies/2011censusgeographies (accessed August 4, 2025).

[bib4] Daniels K.M., Schinasi L.H., Auchincloss A.H., Forrest C.B., Diez Roux A.V. (2021). The built and social neighborhood environment and child obesity: a systematic review of longitudinal studies. Prev. Med..

[bib5] Daniels K.M., Schinasi L.H., Auchincloss A.H., Forrest C.B., Diez Roux A.V. (2021). The built and social neighborhood environment and child obesity: a systematic review of longitudinal studies. Prev. Med..

[bib6] Datar A., Nicosia N., Shier V. (2013). Parent perceptions of neighborhood safety and children's physical activity, sedentary behavior, and obesity: evidence from a national longitudinal study. Am. J. Epidemiol..

[bib7] Davies J., Bailey R., Mizen A., Pouliou T., Fry R., Pedrick-Case R. (2024). Residential mobility amongst children and young people in Wales: a longitudinal study using linked administrative records. Int J Popul Data Sci.

[bib8] Garrett J.K., Rowney F.M., White M.P., Lovell R., Fry R.J., Akbari A. (2023). Visiting nature is associated with lower socioeconomic inequalities in well-being in Wales. Sci. Rep..

[bib9] GBD 2021 Adolescent BMI Collaborators (2025). Global, regional, and national prevalence of child and adolescent overweight and obesity, 1990-2021, with forecasts to 2050: a forecasting study for the global burden of disease study 2021. Lancet.

[bib10] Giles-Corti B., Vernez-Moudon A., Reis R., Turrell G., Dannenberg A.L., Badland H. (2016). City planning and population health: a global challenge. Lancet.

[bib11] Guseman E.H., Sisson S.B., Whipps J., Howe C.A., Byra M.M., Silver L.E. (2022). Neighborhood and family characteristics associated with adiposity and physical activity engagement among preschoolers in a small rural community. Int. J. Environ. Res. Publ. Health.

[bib12] Hirsch J.A., Meyer K.A., Peterson M., Rodriguez D.A., Song Y., Peng K. (2016). Obtaining longitudinal built environment data retrospectively across 25 years in four US cities. Front. Public Health.

[bib13] Hirsch J.A., Meyer K.A., Peterson M., Rodriguez D.A., Song Y., Peng K. (2016). Obtaining longitudinal built environment data retrospectively across 25 years in four US cities. Front. Public Health.

[bib14] Huete A., Didan K., Miura T., Rodriguez E.P., Gao X., Ferreira L.G. (2002). Overview of the radiometric and biophysical performance of the MODIS vegetation indices. Remote Sens. Environ..

[bib15] Jenkin G L., Pearson A L., Bentham G., Day P., Kingham S. (2015). Neighbourhood influences on children's weight-related behaviours and body mass index. AIMS Public Health.

[bib16] Jia P., Cao X., Yang H., Dai S., He P., Huang G. (2020). Green space access in the neighbourhood and childhood obesity. Obes. Rev..

[bib17] Jia P., Zou Y., Wu Z., Zhang D., Wu T., Smith M. (2021). Street connectivity, physical activity, and childhood obesity: a systematic review and meta‐analysis. Obes. Rev..

[bib18] Jones K.H., Ford D.V., Jones C., Dsilva R., Thompson S., Brooks C.J. (2014). A case study of the secure anonymous information linkage (SAIL) gateway: a privacy-protecting remote access system for health-related research and evaluation. J. Biomed. Inf..

[bib19] Jones K.H., Ford D.V., Thompson S., Lyons R. (2019). A profile of the SAIL databank on the UK secure research platform. Int J Popul Data Sci.

[bib20] Kegler M.C., Escoffery C., Alcantara I., Ballard D., Glanz K. (2009). A qualitative examination of home and neighborhood environments for obesity prevention in rural adults. Int. J. Behav. Nutr. Phys. Activ..

[bib21] Ling J., Chen S., Zahry N.R., Kao T.S.A. (2023). Economic burden of childhood overweight and obesity: a systematic review and meta-analysis. Obes. Rev..

[bib22] Lyons R.A., Jones K.H., John G., Brooks C.J., Verplancke J.-P., Ford D.V. (2009). The SAIL databank: linking multiple health and social care datasets. BMC Med. Inf. Decis. Making.

[bib23] Mah S.M., Dasgupta K., Akbari A., Ross N.A., Fry R. (2022). An international comparative study of active living environments and hospitalization for Wales and Canada. SSM Popul. Health.

[bib24] Maitland C., Stratton G., Foster S., Braham R., Rosenberg M. (2013). A place for play? The influence of the home physical environment on children's physical activity and sedentary behaviour. Int. J. Behav. Nutr. Phys. Activ..

[bib25] Malacarne D., Handakas E., Robinson O., Pineda E., Saez M., Chatzi L. (2022). The built environment as determinant of childhood obesity: a systematic literature review. Obes. Rev..

[bib26] Mayor S. (2014). Children living near fast food outlets in England are more likely to be overweight, study shows. BMJ.

[bib27] Miller L.J., Joyce S., Carter S., Yun G. (2014). Associations between childhood obesity and the availability of food outlets in the local environment: a retrospective cross-sectional study. Am. J. Health Promot..

[bib28] Mizen A., Rodgers S., Fry R., Lyons R. (2018). Linking environment and health data to investigate the association between access to unhealthy food and child BMI. Int J Popul Data Sci.

[bib29] Mizen A., Song J., Fry R., Akbari A., Berridge D., Parker S.C. (2019). Longitudinal access and exposure to green-blue spaces and individual-level mental health and well-being: protocol for a longitudinal, population-wide record-linked natural experiment. BMJ Open.

[bib30] Mizen A., Thompson D.A., Watkins A., Akbari A., Garrett J.K., Geary R. (2024). The use of enhanced vegetation index for assessing access to different types of green space in epidemiological studies. J. Expo. Sci. Environ. Epidemiol..

[bib31] Mizen A., Thompson D.A., Watkins A., Akbari A., Garrett J.K., Geary R. (2024). The use of enhanced vegetation index for assessing access to different types of green space in epidemiological studies. J. Expo. Sci. Environ. Epidemiol..

[bib32] Nordbø E.C.A., Raanaas R.K., Nordh H., Aamodt G. (2019). Neighborhood green spaces, facilities and population density as predictors of activity participation among 8-year-olds: a cross-sectional GIS study based on the Norwegian mother and child cohort study. BMC Public Health.

[bib33] Obesity and overweight n.d. https://www.who.int/news-room/fact-sheets/detail/obesity-and-overweight (accessed June 18, 2025).

[bib34] Office for National Statistics (2013).

[bib35] OS MasterMap topography layer, great Britain's landscape vector map data n.d. https://www.ordnancesurvey.co.uk/business-government/products/mastermap-topography (accessed April 21, 2021).

[bib36] OS open greenspace data, products OS n.d. https://www.ordnancesurvey.co.uk/products/os-open-greenspace#get (accessed August 4, 2025).

[bib37] Poole R., Moon G. (2017). What is the association between healthy weight in 4–5-year-old children and spatial access to purposefully constructed play areas?. Health Place.

[bib38] QGIS manual. i.Vi - GRASS 2020. https://grass.osgeo.org/grass78/manuals/i.vi.html. (accessed August 22, 2024).

[bib39] Reimers A.K., Knapp G. (2017). Playground usage and physical activity levels of children based on playground spatial features. J. Publ. Health.

[bib40] Robinson T., Boruff B., Duncan J., Murray K., Schipperijn J., Nathan A. (2024). Understanding variations in the built environment over time to inform longitudinal studies of young children's physical activity behaviour - the BEACHES project. Health Place.

[bib42] Rodgers S.E., Demmler J.C., Dsilva R., Lyons R.A. (2012). Protecting health data privacy while using residence-based environment and demographic data. Health Place.

[bib41] Rodgers S.E., Lyons R.A., Dsilva R., Jones K.H., Brooks C.J., Ford D.V. (2009). Residential anonymous linking fields (RALFs): a novel information infrastructure to study the interaction between the environment and individuals' health. J. Public Health.

[bib43] Sallis J.F., Floyd M.F., Rodríguez D.A., Saelens B.E. (2012). Role of built environments in physical activity, obesity, and cardiovascular disease. Circulation.

[bib44] Schalkwijk A.A.H., Van Der Zwaard B.C., Nijpels G., Elders P.J.M., Platt L. (2018). The impact of greenspace and condition of the neighbourhood on child overweight. Eur. J. Publ. Health.

[bib45] Schalkwijk A.A.H., Van Der Zwaard B.C., Nijpels G., Elders P.J.M., Platt L. (2018). The impact of greenspace and condition of the neighbourhood on child overweight. Eur. J. Publ. Health.

[bib46] Singh G.K., Siahpush M., Kogan M.D. (2010). Neighborhood socioeconomic conditions, built environments, and childhood obesity. Health Aff..

[bib47] Spence J.C., Cutumisu N., Edwards J., Evans J. (2008). Influence of neighbourhood design and access to facilities on overweight among preschool children. Int. J. Pediatr. Obes..

[bib48] Terrón-Pérez M., Molina-García J., Martínez-Bello V.E., Queralt A. (2021). Relationship between the physical environment and physical activity levels in preschool children: a systematic review. Curr. Environ. Health Rep..

[bib49] The Child Measurement Programme for Wales Publication details Title (2017).

[bib50] Thompson D.A., Geary R.S., Rowney F.M., Fry R., Watkins A., Wheeler B.W. (2022). Cohort profile: the green and blue spaces (GBS) and mental health in Wales e-cohort. Int. J. Epidemiol..

[bib51] tmap Tennekes M. (2018). Thematic maps in R. J. Stat. Software.

[bib52] Urie Bronfenbrenner (1979).

[bib53] Van Der Horst K., Oenema A., Ferreira I., Wendel-Vos W., Giskes K., Van Lenthe F. (2007). A systematic review of environmental correlates of obesity-related dietary behaviors in youth. Health Educ. Res..

[bib54] Van Der Zwaard B.C., Schalkwijk A.A.H., Elders P.J.M., Platt L., Nijpels G. (2018). Does environment influence childhood BMI? A longitudinal analysis of children aged 3–11. J. Epidemiol. Community Health.

[bib55] Welsh Government. Schools in Wales DataMapWales n.d. https://datamap.gov.wales/maps/schools-in-wales/(accessed March 27, 2024).

[bib56] Yang S., Chen X., Wang L., Wu T., Fei T., Xiao Q. (2021). Walkability indices and childhood obesity: a review of epidemiologic evidence. Obes. Rev..

