# Supplementary material for: Environmental phenotypes for healthy weight in children using population-based linked environment and health data: a cross-sectional observational study
Source: Health Place. 2026 Jul;100:103681. doi: 10.1016/j.healthplace.2026.103681 (PMC13388830; doi:10.1016/j.healthplace.2026.103681)
Supplement: Multimedia component 1 [file mmc1.docx]

| **Size of greenspace by type, to the nearest 100m2** | | | |
| --- | --- | --- | --- |
| Size nearest park or garden | **Size (m2)** | | |
| Small | ≤15,300 |  |  |
| Medium | 15,400 | - | 76,700 |
| Large | ≥76,800 |  |  |
| Size nearest playing field |  |  |  |
| Small | ≤16,400 |  |  |
| Medium | 16,500 | - | 34,900 |
| Large | ≥35,000 |  |  |
| Size nearest play space |  |  |  |
| Small | ≤500 |  |  |
| Medium | 600 | - | 1300 |
| Large | ≥1,400 |  |  |
| Size nearest tennis court |  |  |  |
| Small | ≤1,700 |  |  |
| Medium | 1,800 | - | 6,200 |
| Large | ≥6,300 |  |  |
| Size nearest other sports facility | |  |  |
| Small | ≤14,300 |  |  |
| Medium | 14,400 | - | 43,800 |
| Large | ≥43,900 |  |  |
|  |  |  |  |

**Supplementary material**
