# Supplementary material for: Environmental phenotypes for healthy weight in children using population-based linked environment and health data: a cross-sectional observational study
Source: Health Place. 2026 Jul;100:103681. doi: 10.1016/j.healthplace.2026.103681 (PMC13388830; doi:10.1016/j.healthplace.2026.103681)
Supplement: Multimedia component 2 [file mmc2.docx]

**Appendix 1**

**Environmental characteristics by phenotype**

| **Profile name** | **Rural, spacious and isolated** | | | | **Suburban** | | | | **Deprived and underserved** | | | | **Deprived and well-served** | | | | **Dense, coastal and well-connected** | | | |
| --- | --- | --- | --- | --- | --- | --- | --- | --- | --- | --- | --- | --- | --- | --- | --- | --- | --- | --- | --- | --- |
| **Number households (%)** | 24,266 | | 14.5 | | 29,324 | | 17.5 | | 39,227 | | 23.4 | | 53,210 | | 31.7 | | 21,762 | | 13.0 | |
| **Characteristic** | Median | (IQR) | Range | | Median | (IQR) | Range | | Median | (IQR) | Range | | Median | (IQR) | Range | | Median | (IQR) | Range | |
| Parks & gardens | 0 | (0) | 0 | 13 | 0 | (0) | 0 | 15 | 0 | (2) | 0 | 15 | 0 | (2) | 0 | 15 | 4 | (7) | 0 | 15 |
| Playing fields | 0 | (0) | 0 | 0 | 2 | (3) | 1 | 13 | 0 | (0) | 0 | 0 | 3 | (4) | 1 | 13 | 1 | (4) | 0 | 13 |
| Play spaces | 0 | (1) | 0 | 7 | 1 | (2) | 0 | 10 | 2 | (2) | 0 | 10 | 2 | (2) | 0 | 10 | 2 | (3) | 0 | 10 |
| Tennis courts | 0 | (0) | 0 | 0 | 0 | (0) | 0 | 0 | 0 | (0) | 0 | 1 | 0 | (0) | 0 | 1 | 0 | (0) | 0 | 1 |
| Other sports facilities | 0 | (0) | 0 | 10 | 0 | (0) | 0 | 13 | 0 | (1) | 0 | 13 | 0 | (2) | 0 | 13 | 1 | (3) | 0 | 13 |
| Total greenspaces | 0 | (1) | 0 | 13 | 4 | (3) | 1 | 25 | 3 | (4) | 0 | 27 | 9 | (6) | 1 | 27 | 12 | (9) | 0 | 27 |
| Bakeries | 0 | (0) | 0 | 2 | 0 | (0) | 0 | 4 | 0 | (0) | 0 | 6 | 0 | (1) | 0 | 6 | 2 | (2) | 0 | 6 |
| Cafes | 0 | (0) | 0 | 4 | 0 | (0) | 0 | 6 | 0 | (1) | 0 | 18 | 0 | (1) | 0 | 18 | 5 | (7) | 0 | 18 |
| Confectioners | 0 | (0) | 0 | 1 | 0 | (0) | 0 | 1 | 0 | (0) | 0 | 3 | 0 | (0) | 0 | 3 | 1 | (1) | 0 | 3 |
| Convenience stores | 0 | (0) | 0 | 8 | 1 | (2) | 0 | 7 | 2 | (2) | 0 | 19 | 2 | (2) | 0 | 19 | 6 | (6) | 0 | 19 |
| Fast food outlets | 0 | (0) | 0 | 10 | 0 | (1) | 0 | 10 | 1 | (3) | 0 | 28 | 2 | (4) | 0 | 28 | 12 | (11) | 0 | 28 |
| Restaurants | 0 | (0) | 0 | 10 | 0 | (0) | 0 | 9 | 0 | (1) | 0 | 22 | 0 | (1) | 0 | 22 | 4 | (8) | 0 | 22 |
| Supermarkets | 0 | (0) | 0 | 3 | 0 | (0) | 0 | 3 | 0 | (1) | 0 | 4 | 0 | (1) | 0 | 4 | 1 | (2) | 0 | 4 |
| Total food outlets | 0 | (0) | 0 | 17 | 1 | (3) | 0 | 25 | 4 | (5) | 0 | 82 | 7 | (7) | 1 | 82 | 32 | (29) | 3 | 82 |
| EVI | 0.4 | (0.2) | 0.2 | 0.8 | 0.3 | (0.1) | 0 | 0.7 | 0.3 | (0.2) | 0 | 0.7 | 0.3 | (0.1) | 0 | 0.7 | 0.2 | (0.2) | 0 | 0.6 |
|  | **Frequency** | **%** |  |  | **Frequency** | **%** |  |  | **Frequency** | **%** |  |  | **Frequency** | **%** |  |  | **Frequency** | **%** |  |  |
| Size nearest Park or Garden |  |  |  |  |  |  |  |  |  |  |  |  |  |  |  |  |  |  |  |  |
| No park or garden | 23,456 | 96.7 | - | - | 26,998 | 92.1 | - | - | 26,102 | 66.5 | - | - | 34,421 | 64.7 | - | - | 4,396 | 20.2 | - | - |
| Small | 104 | 0.4 | - | - | 765 | 2.6 | - | - | 2,722 | 6.9 | - | - | 5,661 | 10.6 | - | - | 4,922 | 22.6 | - | - |
| Medium | 179 | 0.7 | - | - | 663 | 2.3 | - | - | 4,630 | 11.8 | - | - | 6,203 | 11.7 | - | - | 7,193 | 33.1 | - | - |
| Large | 527 | 2.2 | - | - | 898 | 3.1 | - | - | 5,773 | 14.7 | - | - | 6,925 | 13.0 | - | - | 5,251 | 24.1 | - | - |
| Size nearest Playing field |  |  |  |  |  |  |  |  |  |  |  |  |  |  |  |  |  |  |  |  |
| No playing field | 24,266 | 100.0 | - | - | 0 | 0.0 | - | - | 39,227 | 100.0 | - | - | 0 | 0.0 | - | - | 8,787 | 40.4 | - | - |
| Small | 0 | 0.0 | - | - | 12,230 | 41.7 | - | - | 0 | 0.0 | - | - | 13,184 | 24.8 | - | - | 4,116 | 18.9 | - | - |
| Medium | 0 | 0.0 | - | - | 9,044 | 30.8 | - | - | 0 | 0.0 | - | - | 18,987 | 35.7 | - | - | 3,893 | 17.9 | - | - |
| Large | 0 | 0.0 | - | - | 8,050 | 27.5 | - | - | 0 | 0.0 | - | - | 21,039 | 39.5 | - | - | 4,966 | 22.8 | - | - |
| Size nearest play space |  |  |  |  |  |  |  |  |  |  |  |  |  |  |  |  |  |  |  |  |
| No play space | 17,665 | 72.8 | - | - | 9,019 | 30.8 | - | - | 7,461 | 19.0 | - | - | 5,156 | 9.7 | - | - | 3,728 | 17.1 | - | - |
| Small | 2,953 | 12.2 | - | - | 7,969 | 27.2 | - | - | 12,438 | 31.7 | - | - | 16,765 | 31.5 | - | - | 4,037 | 18.6 | - | - |
| Medium | 2,040 | 8.4 | - | - | 7,097 | 24.2 | - | - | 9,359 | 23.9 | - | - | 15,961 | 30.0 | - | - | 6,741 | 31.0 | - | - |
| Large | 1,608 | 6.6 | - | - | 5,239 | 17.9 | - | - | 9,969 | 25.4 | - | - | 15,328 | 28.8 | - | - | 7,256 | 33.3 | - | - |
| Size nearest tennis court |  |  |  |  |  |  |  |  |  |  |  |  |  |  |  |  |  |  |  |  |
| No tennis court | 24,266 | 100.0 | - | - | 29,324 | 100.0 | - | - | 38,997 | 99.4 | - | - | 52,807 | 99.2 | - | - | 20,707 | 95.2 | - | - |
| Small | 0 | 0.0 | - | - | 0 | 0.0 | - | - | 46 | 0.1 | - | - | 209 | 0.4 | - | - | 237 | 1.1 | - | - |
| Medium | 0 | 0.0 | - | - | 0 | 0.0 | - | - | 13 | 0.0 | - | - | 153 | 0.3 | - | - | 589 | 2.7 | - | - |
| Large | 0 | 0.0 | - | - | 0 | 0.0 | - | - | 171 | 0.4 | - | - | 41 | 0.1 | - | - | 229 | 1.1 | - | - |
| Size nearest other sports facility |  |  |  |  |  |  |  |  |  |  |  |  |  |  |  |  |  |  |  |  |
| No other sports facility | 23,900 | 98.5 | - | - | 27,537 | 93.9 | - | - | 26,849 | 68.4 | - | - | 31,184 | 58.6 | - | - | 9,580 | 44.0 | - | - |
| Small | 146 | 0.6 | - | - | 817 | 2.8 | - | - | 4,770 | 12.2 | - | - | 7,723 | 14.5 | - | - | 4,728 | 21.7 | - | - |
| Medium | 128 | 0.5 | - | - | 425 | 1.4 | - | - | 3,375 | 8.6 | - | - | 6,457 | 12.1 | - | - | 3,001 | 13.8 | - | - |
| Large | 92 | 0.4 | - | - | 545 | 1.9 | - | - | 4,233 | 10.8 | - | - | 7,846 | 14.7 | - | - | 4,453 | 20.5 | - | - |
| Garden size (m^2^) |  |  |  |  |  |  |  |  |  |  |  |  |  |  |  |  |  |  |  |  |
| No Garden | 1,356 | 5.6 | - | - | 911 | 3.1 | - | - | 704 | 1.8 | - | - | 746 | 1.4 | - | - | 550 | 2.5 | - | - |
| 0-99.9 | 2,198 | 9.1 | - | - | 4,672 | 15.9 | - | - | 9,352 | 23.8 | - | - | 13,970 | 26.3 | - | - | 11,134 | 51.2 | - | - |
| 100-199.9 | 5,016 | 20.7 | - | - | 9,679 | 33.0 | - | - | 13,558 | 34.6 | - | - | 18,030 | 33.9 | - | - | 4,375 | 20.1 | - | - |
| 200-299.9 | 3,961 | 16.3 | - | - | 6,385 | 21.8 | - | - | 7,813 | 19.9 | - | - | 10,704 | 20.1 | - | - | 1,922 | 8.8 | - | - |
| 300-399.9 | 2,179 | 9.0 | - | - | 2,701 | 9.2 | - | - | 2,861 | 7.3 | - | - | 3,672 | 6.9 | - | - | 705 | 3.2 | - | - |
| 400-499.9 | 1,484 | 6.1 | - | - | 1,275 | 4.3 | - | - | 1,180 | 3.0 | - | - | 1,357 | 2.6 | - | - | 305 | 1.4 | - | - |
| 500+ | 6,823 | 28.1 | - | - | 2,507 | 8.5 | - | - | 1,944 | 5.0 | - | - | 1,859 | 3.5 | - | - | 463 | 2.1 | - | - |
| Missing | 1,249 | 5.1 | - | - | 1,194 | 4.1 | - | - | 1,815 | 4.6 | - | - | 2,872 | 5.4 | - | - | 2,308 | 10.6 | - | - |
| Nearest primary school |  |  |  |  |  |  |  |  |  |  |  |  |  |  |  |  |  |  |  |  |
| Hyper-local | 1,406 | 5.8 | - | - | 3,323 | 11.3 | - | - | 4,527 | 11.5 | - | - | 9,038 | 17.0 | - | - | 4,877 | 22.4 | - | - |
| Local | 2,507 | 10.3 | - | - | 7,546 | 25.7 | - | - | 11,140 | 28.4 | - | - | 19,083 | 35.9 | - | - | 9,071 | 41.7 | - | - |
| Neighbourhood | 2,880 | 11.9 | - | - | 8,072 | 27.5 | - | - | 10,520 | 26.8 | - | - | 15,345 | 28.8 | - | - | 4,903 | 22.5 | - | - |
| >900m | 17,445 | 71.9 | - | - | 10,344 | 35.3 | - | - | 12,981 | 33.1 | - | - | 9,655 | 18.1 | - | - | 2,814 | 12.9 | - | - |
| Missing | 28 | 0.1 | - | - | 39 | 0.1 | - | - | 59 | 0.2 | - | - | 89 | 0.2 | - | - | 97 | 0.4 | - | - |
| Average distance to coast (miles) |  |  |  |  |  |  |  |  |  |  |  |  |  |  |  |  |  |  |  |  |
| <1 | 356 | 1.5 | - | - | 323 | 1.1 | - | - | 891 | 2.3 | - | - | 1,080 | 2.0 | - | - | 1,392 | 6.4 | - | - |
| 1 | 1,961 | 8.1 | - | - | 2,996 | 10.2 | - | - | 4,442 | 11.3 | - | - | 5,652 | 10.6 | - | - | 4,074 | 18.7 | - | - |
| 2 | 1,495 | 6.2 | - | - | 1,840 | 6.3 | - | - | 3,366 | 8.6 | - | - | 3,058 | 5.7 | - | - | 3,408 | 15.7 | - | - |
| 3 | 1,333 | 5.5 | - | - | 2,360 | 8.0 | - | - | 2,611 | 6.7 | - | - | 4,559 | 8.6 | - | - | 1,630 | 7.5 | - | - |
| 4 | 2,101 | 8.7 | - | - | 1,793 | 6.1 | - | - | 4,146 | 10.6 | - | - | 3,785 | 7.1 | - | - | 1,888 | 8.7 | - | - |
| 5 | 1,035 | 4.3 | - | - | 1,952 | 6.7 | - | - | 2,400 | 6.1 | - | - | 3,159 | 5.9 | - | - | 2,026 | 9.3 | - | - |
| 6 | 2,017 | 8.3 | - | - | 2,274 | 7.8 | - | - | 1,964 | 5.0 | - | - | 3,685 | 6.9 | - | - | 651 | 3.0 | - | - |
| 7-10 | 4,050 | 16.7 | - | - | 4,482 | 15.3 | - | - | 4,591 | 11.7 | - | - | 5,772 | 10.8 | - | - | 1,606 | 7.4 | - | - |
| 11-14 | 3,012 | 12.4 | - | - | 3,737 | 12.7 | - | - | 3,574 | 9.1 | - | - | 6,942 | 13.0 | - | - | 1,314 | 6.0 | - | - |
| 15+ | 6,906 | 28.5 | - | - | 7,567 | 25.8 | - | - | 11,242 | 28.7 | - | - | 15,518 | 29.2 | - | - | 3,773 | 17.3 | - | - |
| Walkability index |  |  |  |  |  |  |  |  |  |  |  |  |  |  |  |  |  |  |  |  |
| 1 - Most Walkable | 15,701 | 64.7 | - | - | 12,378 | 42.21 | - | - | 7,405 | 18.88 | - | - | 8,361 | 15.71 | - | - | 1,111 | 5.11 | - | - |
| 2 | 3,057 | 12.6 | - | - | 6,437 | 21.95 | - | - | 11,661 | 29.73 | - | - | 16,847 | 31.66 | - | - | 7,003 | 32.18 | - | - |
| 3 | 370 | 1.52 | - | - | 1,696 | 5.78 | - | - | 6,109 | 15.57 | - | - | 9,859 | 18.53 | - | - | 6,453 | 29.65 | - | - |
| 4 | 5,138 | 21.17 | - | - | 8,803 | 30.02 | - | - | 13,927 | 35.5 | - | - | 17,666 | 33.2 | - | - | 3,490 | 16.04 | - | - |
| 5 - Least walkable | - | - | - | - | 10 | 0.03 | - | - | 125 | 0.32 | - | - | 477 | 0.9 | - | - | 3,705 | 17 | - | - |
| House type |  |  |  |  |  |  |  |  |  |  |  |  |  |  |  |  |  |  |  |  |
| Detached | 11,516 | 47.5 | - | - | 7,262 | 24.8 | - | - | 7,248 | 18.5 | - | - | 5,181 | 9.7 | - | - | 1,267 | 5.8 | - | - |
| Self-Contained Flat | 206 | 0.8 | - | - | 567 | 1.9 | - | - | 1,083 | 2.8 | - | - | 1,725 | 3.2 | - | - | 1,562 | 7.2 | - | - |
| Semi-Detached | 5,543 | 22.8 | - | - | 10,304 | 35.1 | - | - | 11,458 | 29.2 | - | - | 17,968 | 33.8 | - | - | 3,552 | 16.3 | - | - |
| Terraced | 3,733 | 15.4 | - | - | 8,205 | 28.0 | - | - | 14,286 | 36.4 | - | - | 23,199 | 43.6 | - | - | 13,119 | 60.3 | - | - |
| Other | 84 | 0.3 | - | - | 20 | 0.1 | - | - | 18 | 0.0 | - | - | 13 | 0.0 | - | - | 68 | 0.3 | - | - |
| Missing | 3,184 | 13.1 | - | - | 2,966 | 10.1 | - | - | 5,134 | 13.1 | - | - | 5,124 | 9.6 | - | - | 2,194 | 10.1 | - | - |
| Urban Rural classification |  |  |  |  |  |  |  |  |  |  |  |  |  |  |  |  |  |  |  |  |
| City & town less sparse | 9,328 | 38.4 | - | - | 17,509 | 59.7 | - | - | 31,209 | 79.6 | - | - | 41,737 | 78.4 | - | - | 18,385 | 84.5 | - | - |
| City & town sparse | 271 | 1.1 | - | - | 803 | 2.7 | - | - | 477 | 1.2 | - | - | 770 | 1.4 | - | - | 660 | 3.0 | - | - |
| Town & fringe less sparse | 2,904 | 12.0 | - | - | 5,741 | 19.6 | - | - | 4,561 | 11.6 | - | - | 8,384 | 15.8 | - | - | 1,298 | 6.0 | - | - |
| Town & fringe sparse | 751 | 3.1 | - | - | 682 | 2.3 | - | - | 1,090 | 2.8 | - | - | 1,763 | 3.3 | - | - | 1,323 | 6.1 | - | - |
| Village less sparse | 4,664 | 19.2 | - | - | 3,047 | 10.4 | - | - | 809 | 2.1 | - | - | 274 | 0.5 | - | - | 14 | 0.1 | - | - |
| Village sparse | 6,348 | 26.2 | - | - | 1,542 | 5.3 | - | - | 1,081 | 2.8 | - | - | 282 | 0.5 | - | - | 82 | 0.4 | - | - |
| WIMD^1^ |  |  |  |  |  |  |  |  |  |  |  |  |  |  |  |  |  |  |  |  |
| 1 - Most deprived | 1,032 | 4.3 | - | - | 4,548 | 15.5 | - | - | 10,968 | 28.0 | - | - | 18,366 | 34.5 | - | - | 7,049 | 32.4 | - | - |
| 2 | 3,124 | 12.9 | - | - | 6,029 | 20.6 | - | - | 7,887 | 20.1 | - | - | 12,705 | 23.9 | - | - | 5,740 | 26.4 | - | - |
| 3 | 5,873 | 24.2 | - | - | 5,387 | 18.4 | - | - | 5,691 | 14.5 | - | - | 9,893 | 18.6 | - | - | 3,986 | 18.3 | - | - |
| 4 | 7,725 | 31.8 | - | - | 6,795 | 23.2 | - | - | 5,151 | 13.1 | - | - | 6,862 | 12.9 | - | - | 3,037 | 14.0 | - | - |
| 5 - Least deprived | 6,512 | 26.8 | - | - | 6,565 | 22.4 | - | - | 9,530 | 24.3 | - | - | 5,384 | 10.1 | - | - | 1,950 | 9.0 | - | - |
| WIMD Income |  |  |  |  |  |  |  |  |  |  |  |  |  |  |  |  |  |  |  |  |
| 1 - Most deprived | 2,555 | 10.5 | - | - | 4,270 | 14.6 | - | - | 7,992 | 20.4 | - | - | 10,759 | 20.2 | - | - | 8,943 | 41.1 | - | - |
| 2 | 3,720 | 15.3 | - | - | 5,557 | 19.0 | - | - | 9,024 | 23.0 | - | - | 11,488 | 21.6 | - | - | 4,590 | 21.1 | - | - |
| 3 | 5,049 | 20.8 | - | - | 5,800 | 19.8 | - | - | 7,936 | 20.2 | - | - | 11,561 | 21.7 | - | - | 3,454 | 15.9 | - | - |
| 4 | 6,611 | 27.2 | - | - | 6,989 | 23.8 | - | - | 7,368 | 18.8 | - | - | 9,986 | 18.8 | - | - | 1,892 | 8.7 | - | - |
| 5 - Least deprived | 6,331 | 26.1 | - | - | 6,708 | 22.9 | - | - | 6,907 | 17.6 | - | - | 9,416 | 17.7 | - | - | 2,883 | 13.2 | - | - |
| WIMD Employment |  |  |  |  |  |  |  |  |  |  |  |  |  |  |  |  |  |  |  |  |
| 1 - Most deprived | 1,161 | 4.8 | - | - | 4,933 | 16.8 | - | - | 11,251 | 28.7 | - | - | 17,456 | 32.8 | - | - | 5,678 | 26.1 | - | - |
| 2 | 2,442 | 10.1 | - | - | 5,961 | 20.3 | - | - | 7,650 | 19.5 | - | - | 14,120 | 26.5 | - | - | 6,302 | 29.0 | - | - |
| 3 | 4,156 | 17.1 | - | - | 6,189 | 21.1 | - | - | 6,926 | 17.7 | - | - | 11,468 | 21.6 | - | - | 4,766 | 21.9 | - | - |
| 4 | 7,719 | 31.8 | - | - | 5,880 | 20.1 | - | - | 6,100 | 15.6 | - | - | 6,308 | 11.9 | - | - | 3,075 | 14.1 | - | - |
| 5 - Least deprived | 8,788 | 36.2 | - | - | 6,361 | 21.7 | - | - | 7,300 | 18.6 | - | - | 3,858 | 7.3 | - | - | 1,941 | 8.9 | - | - |
| WIMD Health |  |  |  |  |  |  |  |  |  |  |  |  |  |  |  |  |  |  |  |  |
| 1 - Most deprived | 1,228 | 5.1 | - | - | 5,364 | 18.3 | - | - | 11,178 | 28.5 | - | - | 18,077 | 34.0 | - | - | 5,971 | 27.4 | - | - |
| 2 | 2,299 | 9.5 | - | - | 6,198 | 21.1 | - | - | 8,038 | 20.5 | - | - | 14,058 | 26.4 | - | - | 5,676 | 26.1 | - | - |
| 3 | 3,625 | 14.9 | - | - | 6,382 | 21.8 | - | - | 7,070 | 18.0 | - | - | 11,756 | 22.1 | - | - | 5,195 | 23.9 | - | - |
| 4 | 8,317 | 34.3 | - | - | 5,867 | 20.0 | - | - | 6,243 | 15.9 | - | - | 5,968 | 11.2 | - | - | 2,832 | 13.0 | - | - |
| 5 - Least deprived | 8,797 | 36.3 | - | - | 5,513 | 18.8 | - | - | 6,698 | 17.1 | - | - | 3,351 | 6.3 | - | - | 2,088 | 9.6 | - | - |
| WIMD Education |  |  |  |  |  |  |  |  |  |  |  |  |  |  |  |  |  |  |  |  |
| 1 - Most deprived | 1,225 | 5.0 | - | - | 5,246 | 17.9 | - | - | 11,270 | 28.7 | - | - | 19,057 | 35.8 | - | - | 5,042 | 23.2 | - | - |
| 2 | 2,506 | 10.3 | - | - | 5,891 | 20.1 | - | - | 8,058 | 20.5 | - | - | 12,881 | 24.2 | - | - | 6,257 | 28.8 | - | - |
| 3 | 4,351 | 17.9 | - | - | 6,500 | 22.2 | - | - | 6,224 | 15.9 | - | - | 11,066 | 20.8 | - | - | 4,560 | 21.0 | - | - |
| 4 | 8,792 | 36.2 | - | - | 5,515 | 18.8 | - | - | 6,351 | 16.2 | - | - | 6,132 | 11.5 | - | - | 3,075 | 14.1 | - | - |
| 5 - Least deprived | 7,392 | 30.5 | - | - | 6,172 | 21.0 | - | - | 7,324 | 18.7 | - | - | 4,074 | 7.7 | - | - | 2,828 | 13.0 | - | - |
| WIMD Access |  |  |  |  |  |  |  |  |  |  |  |  |  |  |  |  |  |  |  |  |
| 1 - Most deprived | 12,469 | 51.4 | - | - | 6,181 | 21.1 | - | - | 5,295 | 13.5 | - | - | 4,600 | 8.6 | - | - | 1,299 | 6.0 | - | - |
| 2 | 2,824 | 11.6 | - | - | 7,334 | 25.0 | - | - | 9,090 | 23.2 | - | - | 13,286 | 25.0 | - | - | 3,942 | 18.1 | - | - |
| 3 | 3,174 | 13.1 | - | - | 6,299 | 21.5 | - | - | 8,046 | 20.5 | - | - | 13,323 | 25.0 | - | - | 4,975 | 22.9 | - | - |
| 4 | 2,927 | 12.1 | - | - | 5,260 | 17.9 | - | - | 8,426 | 21.5 | - | - | 11,478 | 21.6 | - | - | 5,541 | 25.5 | - | - |
| 5 - Least deprived | 2,872 | 11.8 | - | - | 4,250 | 14.5 | - | - | 8,370 | 21.3 | - | - | 10,523 | 19.8 | - | - | 6,005 | 27.6 | - | - |
| WIMD Houing |  |  |  |  |  |  |  |  |  |  |  |  |  |  |  |  |  |  |  |  |
| 1 - Most deprived | 4,692 | 19.3 | - | - | 2,889 | 9.9 | - | - | 4,425 | 11.3 | - | - | 8,735 | 16.4 | - | - | 10,660 | 49.0 | - | - |
| 2 | 4,134 | 17.0 | - | - | 4,792 | 16.3 | - | - | 8,285 | 21.1 | - | - | 13,806 | 25.9 | - | - | 4,393 | 20.2 | - | - |
| 3 | 4,302 | 17.7 | - | - | 6,547 | 22.3 | - | - | 8,431 | 21.5 | - | - | 13,002 | 24.4 | - | - | 3,170 | 14.6 | - | - |
| 4 | 4,414 | 18.2 | - | - | 7,461 | 25.4 | - | - | 8,032 | 20.5 | - | - | 12,081 | 22.7 | - | - | 2,589 | 11.9 | - | - |
| 5 - Least deprived | 6,724 | 27.7 | - | - | 7,635 | 26.0 | - | - | 10,054 | 25.6 | - | - | 5,586 | 10.5 | - | - | 950 | 4.4 | - | - |
| WIMD Community Safety |  |  |  |  |  |  |  |  |  |  |  |  |  |  |  |  |  |  |  |  |
| 1 - Most deprived | 1,521 | 6.3 | - | - | 3,184 | 10.9 | - | - | 8,582 | 21.9 | - | - | 15,715 | 29.5 | - | - | 9,006 | 41.4 | - | - |
| 2 | 1,914 | 7.9 | - | - | 5,863 | 20.0 | - | - | 8,140 | 20.8 | - | - | 14,881 | 28.0 | - | - | 5,534 | 25.4 | - | - |
| 3 | 3,391 | 14.0 | - | - | 6,319 | 21.5 | - | - | 7,867 | 20.1 | - | - | 11,554 | 21.7 | - | - | 4,393 | 20.2 | - | - |
| 4 | 6,505 | 26.8 | - | - | 7,291 | 24.9 | - | - | 8,183 | 20.9 | - | - | 8,002 | 15.0 | - | - | 2,199 | 10.1 | - | - |
| 5 - Least deprived | 10,935 | 45.1 | - | - | 6,667 | 22.7 | - | - | 6,455 | 16.5 | - | - | 3,058 | 5.7 | - | - | 630 | 2.9 | - | - |
| WIMD Physical Environment |  |  |  |  |  |  |  |  |  |  |  |  |  |  |  |  |  |  |  |  |
| 1 - Most deprived | 2,555 | 10.5 | - | - | 4,270 | 14.6 | - | - | 7,992 | 20.4 | - | - | 10,759 | 20.2 | - | - | 8,943 | 41.1 | - | - |
| 2 | 3,720 | 15.3 | - | - | 5,557 | 19.0 | - | - | 9,024 | 23.0 | - | - | 11,488 | 21.6 | - | - | 4,590 | 21.1 | - | - |
| 3 | 5,049 | 20.8 | - | - | 5,800 | 19.8 | - | - | 7,936 | 20.2 | - | - | 11,561 | 21.7 | - | - | 3,454 | 15.9 | - | - |
| 4 | 6,611 | 27.2 | - | - | 6,989 | 23.8 | - | - | 7,368 | 18.8 | - | - | 9,986 | 18.8 | - | - | 1,892 | 8.7 | - | - |
| 5 - Least deprived | 6,331 | 26.1 | - | - | 6,708 | 22.9 | - | - | 6,907 | 17.6 | - | - | 9,416 | 17.7 | - | - | 2,883 | 13.2 | - | - |
| ^1^WIMD – Welsh Index of Multiple Deprivation (measure of small-area geography) | | | | | | | | | | | | | | | | | | | |  |
|  |  |  |  |  |  |  |  |  |  |  |  |  |  |  |  |  |  |  |  |  |

**Appendix 2**

**Latent class analysis - model fit statistics**

The model with nine classes failed to converge. The model with eight classes had the lowest AIC value, however the six, seven and eight class models all had classes that contained less than 10% of the sample. The five class model was selected as the best fitting model.

| Class | n | ll(null) | ll(model) | df | AIC | BIC |
| --- | --- | --- | --- | --- | --- | --- |
| threeclass | 167,789 | . | 1973838 | 107 | 3947890 | 3948963 |
| fourclass | 167,789 | . | 1926573 | 143 | 3853432 | 3854866 |
| fiveclass | 167,789 | . | 1857564 | 178 | 3715485 | 3717270 |
| sixclass | 167,789 | . | 1818534 | 215 | 3637498 | 3639654 |
| sevenclass | 167,789 | . | 1760539 | 251 | 3521581 | 3524098 |
| eightclass | 167,789 | . | 1739441 | 287 | 3479456 | 3482335 |
